# Fire dragon cupping therapy for primary dysmenorrhea: a systematic review of available clinical evidence and research gaps

**DOI:** 10.3389/fendo.2026.1885285

**Published:** 2026-09-29

**Authors:** Xinyu Zhang, Qin Zhou, Jie Yu, Min Li, Huiling Zhong

**Affiliations:** 1 Gynecology Department, Tongxiang Hospital of Traditional Chinese Medicine, Jiaxing, Zhejiang, China; 2 Emergency Department, Tongxiang Hospital of Traditional Chinese Medicine, Jiaxing, Zhejiang, China

**Keywords:** complementary medicine, evidence gap, menstrual pain, narrative synthesis, traditional Chinese medicine

## Abstract

**Background:**

Fire Dragon Cupping Therapy (FDCT) combines negative-pressure cupping with controlled thermal stimulation, moving-cup manipulation, and acupoint stimulation, and is increasingly used for primary dysmenorrhea (PD), but its clinical evidence remains uncertain.

**Objective:**

To identify randomized controlled trials (RCTs) of FDCT for PD and, when no eligible RCTs were found, to map direct non-randomized and indirect contextual evidence.

**Methods:**

PubMed/MEDLINE, Embase, Cochrane CENTRAL, CNKI, Wanfang, VIP, ClinicalTrials.gov, and the WHO International Clinical Trials Registry Platform were searched from inception to March 2026. Eligible primary studies were evaluating FDCT alone or as an adjunct in women with PD. Direct non-randomized FDCT studies and indirect studies of related cupping or Traditional Chinese Medicine (TCM) interventions were summarized separately and were not used to estimate FDCT effectiveness.

**Results:**

Among 890 records, 248 remained after duplicate removal and 80 full-text reports were assessed; no RCT met the primary eligibility criteria. One retrospective comparative study of 112 patients provided the most informative direct evidence: 58 received FDCT plus Shaofu Zhuyu Decoction and 54 received the decoction alone. Favorable post-treatment pain, TCM syndrome, uterine hemodynamic, endothelin-1, and nitric oxide findings were reported (all P < 0.05), but non-randomized allocation and the combined intervention precluded attribution to FDCT. Additional direct or contextual reports were heterogeneous and methodologically limited. Certainty of evidence was very low, and safety reporting was insufficient.

**Conclusion:**

Current evidence is insufficient to establish the efficacy or safety of FDCT for PD. Adequately powered multicenter RCTs using standardized FDCT protocols, validated outcomes, appropriate comparators, and systematic adverse-event monitoring are required.

## 1 Introduction

Primary dysmenorrhea (PD) is recurrent cramping pelvic pain occurring immediately before or during menstruation in the absence of identifiable pelvic pathology (1). Symptoms may include nausea, headache, fatigue, diarrhea and back pain and can substantially impair daily activities, academic performance, and quality of life (2). PD is highly prevalent among adolescents and young women, and systematic evidence indicates a substantial burden of school or work absenteeism and reduced performance (3, 4).

The pathophysiology of PD is closely related to increased endometrial prostaglandin production during menstruation. Excess prostaglandin F2α and E2 promote uterine hypercontractility, vasoconstriction, reduced uterine perfusion, and ischemic pain (5). Non-steroidal anti-inflammatory drugs (NSAIDs), which inhibit cyclooxygenase-mediated prostaglandin synthesis, are first-line pharmacological therapy, while hormonal contraceptives are also used to suppress ovulation and reduce endometrial prostaglandin production (2, 6). Nevertheless, some patients have incomplete pain relief, contraindications, or treatment-related adverse effects, supporting continued investigation of complementary approaches (7).

Fire Dragon Cupping Therapy (FDCT) is a specialized cupping-based intervention combining negative-pressure suction with controlled thermal stimulation and manual manipulation. Unlike conventional stationary dry cupping, FDCT may incorporate heated cups or moxa-generated warmth, sliding or walking-cup movements, scraping, kneading, massage, and stimulation of selected meridians and acupoints. Available clinical descriptions commonly apply treatment to the back, lumbosacral region, lower abdomen, and baliao acupoints. Its distinguishing feature is therefore the combined use of suction, heat, movement, and acupoint stimulation rather than suction alone (8, 9).

From a Traditional Chinese Medicine (TCM) perspective, FDCT is intended to warm the meridians, disperse cold, promote Qi and blood circulation, and reduce stagnation. Biomedical hypotheses include modulation of pain perception, local circulation, uterine perfusion, and vascular mediators. However, these mechanisms are supported mainly by indirect evidence from conventional cupping, moxibustion, acupuncture, herbal treatment, or multimodal interventions (8, 10, 11). Biological plausibility should therefore not be interpreted as evidence of FDCT clinical effectiveness.

Because publications on FDCT include non-randomized studies, mixed interventions, related but non-equivalent therapies, reviews, and trial registrations, these evidence types cannot be combined to establish efficacy. This review therefore aimed to identify and critically appraise RCTs of FDCT for PD. Meta-analysis was planned only if sufficiently comparable randomized evidence was available. When no eligible RCTs were identified, direct non-randomized and indirect evidence was mapped separately to characterize the evidence gap and inform future research.

## 2 Methodology

### 2.1 Study design and reporting

This systematic review was conducted and reported in accordance with PRISMA 2020 (12). The primary review question was whether FDCT, used alone or as an adjunctive intervention, is effective and safe for women with PD. A quantitative synthesis was prespecified only if at least two clinically and methodologically comparable randomized controlled trials (RCTs) were identified. Because no eligible RCTs were found, no meta-analysis, pooled effect estimate, statistical subgroup analysis, sensitivity analysis, or publication-bias analysis was performed.

### 2.2 Eligibility criteria

Randomized or cluster-RCTs were eligible when they enrolled women aged 18–45 years with PD diagnosed using recognized clinical criteria, including recurrent menstrual pain without identifiable pelvic pathology, and evaluated FDCT alone or FDCT added to a co-intervention. TCM syndrome classifications such as cold coagulation and blood stasis were permitted when reported. Eligible comparators included sham FDCT, no intervention, usual care, pharmacological therapy, herbal therapy, or the same co-intervention without FDCT. Studies were required to report at least one relevant clinical outcome, including pain intensity, dysmenorrhea symptom severity, quality of life, functional impairment, treatment response, recurrence, uterine hemodynamic parameters, biochemical outcomes, or adverse events.

Studies of secondary dysmenorrhea, non-randomized designs, case reports, reviews, protocols, conference abstracts without sufficient data, animal studies, and studies without a distinct FDCT intervention were excluded from the primary systematic review.

### 2.3 Contextual evidence

After no eligible RCTs were identified, direct non-randomized FDCT studies were examined separately as contextual evidence. Studies of conventional cupping, moxibustion, acupuncture, acupressure, herbal treatment, or multimodal TCM interventions were classified as indirect evidence. Reviews, protocols, and ongoing clinical trials were described separately and were not combined with completed primary clinical studies. Contextual evidence was used only to characterize the research landscape, identify potential mechanisms, and research gaps and not to establish FDCT efficacy or safety (13, 14).

### 2.4 Information sources and search strategy

PubMed/MEDLINE, Embase, Cochrane CENTRAL, CNKI, Wanfang, and VIP were searched from database inception to March 2026. ClinicalTrials.gov and the WHO International Clinical Trials Registry Platform were searched for ongoing or unpublished studies. No language or publication-status restriction was applied. Reference lists of potentially relevant studies and reviews were also examined. Search terms combined controlled vocabulary and free-text terms for ‘Fire Dragon Cupping’, ‘Huo Long Guan’, ‘Fire Dragon Moxibustion’, ‘primary dysmenorrhea’, ‘dysmenorrhea’, ‘randomized controlled trial’, and ‘RCT’ using Boolean operators AND and OR.

### 2.5 Study selection

All retrieved records were imported into reference-management software and duplicates were removed before screening. Two reviewers independently screened titles and abstracts and then assessed potentially eligible full-text reports. Disagreements were resolved by discussion and, when necessary, consultation with a third reviewer. Final eligibility decisions were reached by consensus. A formal kappa statistic was not prospectively recorded and this is acknowledged as a methodological limitation.

### 2.6 Data extraction

Two reviewers independently extracted information using a standardized data-extraction form. Extracted variables included the complete reference, country, study design and recruitment setting, participant characteristics, sample size, diagnostic criteria, intervention components, treatment frequency and duration, comparator, follow-up period, outcome measures, numerical findings, adverse events, and funding information. Direct FDCT evidence and indirect contextual evidence were recorded separately to avoid treating clinically and methodologically non-equivalent interventions as a single evidence base. Discrepancies in extracted information were resolved by discussion and consensus.

### 2.7 Risk-of-bias assessment and certainty of evidence

Because no randomized controlled trial met the primary eligibility criteria, the Cochrane Risk of Bias 2 (RoB 2) tool was not applied. The most informative direct retrospective comparative study was appraised using domains consistent with the Risk Of Bias In Non-randomized Studies of Interventions (ROBINS-I) framework. Particular attention was paid to non-random treatment allocation, potential confounding, participant selection, classification of interventions, deviations from intended interventions, missing data, outcome measurement, and selective reporting. The study was judged to have a serious overall risk of bias because treatment selection could have reflected patient or clinician preference, residual confounding could not be excluded, FDCT was administered with an active herbal treatment, and blinding was not reported. Certainty was considered very low for pain, dysmenorrhea symptoms, uterine hemodynamic outcomes, biochemical markers, and safety because of serious risk of bias, indirectness arising from combined or non-FDCT interventions, imprecision arising from the limited direct evidence, heterogeneity across contextual sources, and the inability to assess publication bias.

### 2.8 Outcome measures and data synthesis

The primary outcome was menstrual pain intensity measured with a validated pain scale such as the Visual Analog Scale (VAS). Secondary outcomes included dysmenorrhea symptom or TCM syndrome scores, quality of life and functional impairment where reported, treatment response and recurrence, uterine artery resistance index (RI) and pulsatility index (PI), biochemical markers including serum endothelin-1 (ET-1) and nitric oxide (NO), and adverse events such as erythema, burns, infection, or discomfort. Eligible randomized controlled trials were intended to form the primary synthesis. Because no eligible trial was identified, the primary result was reported as the absence of randomized evidence and no pooled analysis was undertaken. Direct non-randomized FDCT studies were summarized separately using their reported sample sizes, interventions, comparators, outcomes, and statistical findings. Indirect evidence was grouped according to intervention type and study design but was not pooled or used to infer the magnitude of an FDCT treatment effect. Terms implying efficacy, superiority, or safety were avoided unless explicitly attributed to an individual source and accompanied by its methodological limitations.

### 2.9 FDCT intervention characteristics

In the available clinical descriptions, FDCT generally used heated glass cups to provide negative-pressure suction and thermal stimulation, often combined with moving- or sliding-cup manipulation and stimulation of selected lumbosacral, abdominal, meridian, or acupoint regions. Protocols varied in heat exposure, session duration, treatment frequency, timing relative to menstruation, number of menstrual cycles, and use of co-interventions such as Shaofu Zhuyu Decoction. These details were extracted as characteristics of the available interventions and were not treated as a standardized review protocol.

## 3 Results

### 3.1 Study selection

The database and registry searches identified 890 records. After 642 duplicate records were removed, 248 unique records remained for title and abstract screening. Of these, 167 were excluded and 81 reports were sought for full-text retrieval. One report could not be retrieved, leaving 80 full-text reports for assessment against the predefined randomized-trial eligibility criteria. None met the criteria for the primary systematic review; therefore, no randomized controlled trial evaluating FDCT for PD was included and no quantitative meta-analysis was performed (**Figure 1**). Full-text reports were excluded because of non-randomized design, absence of an FDCT-specific intervention, mixed interventions without an isolatable FDCT effect, inappropriate publication type, or absence of completed clinical outcome data. A limited body of direct non-randomized and contextual evidence was retained for separate narrative analysis.

**Figure 1:**
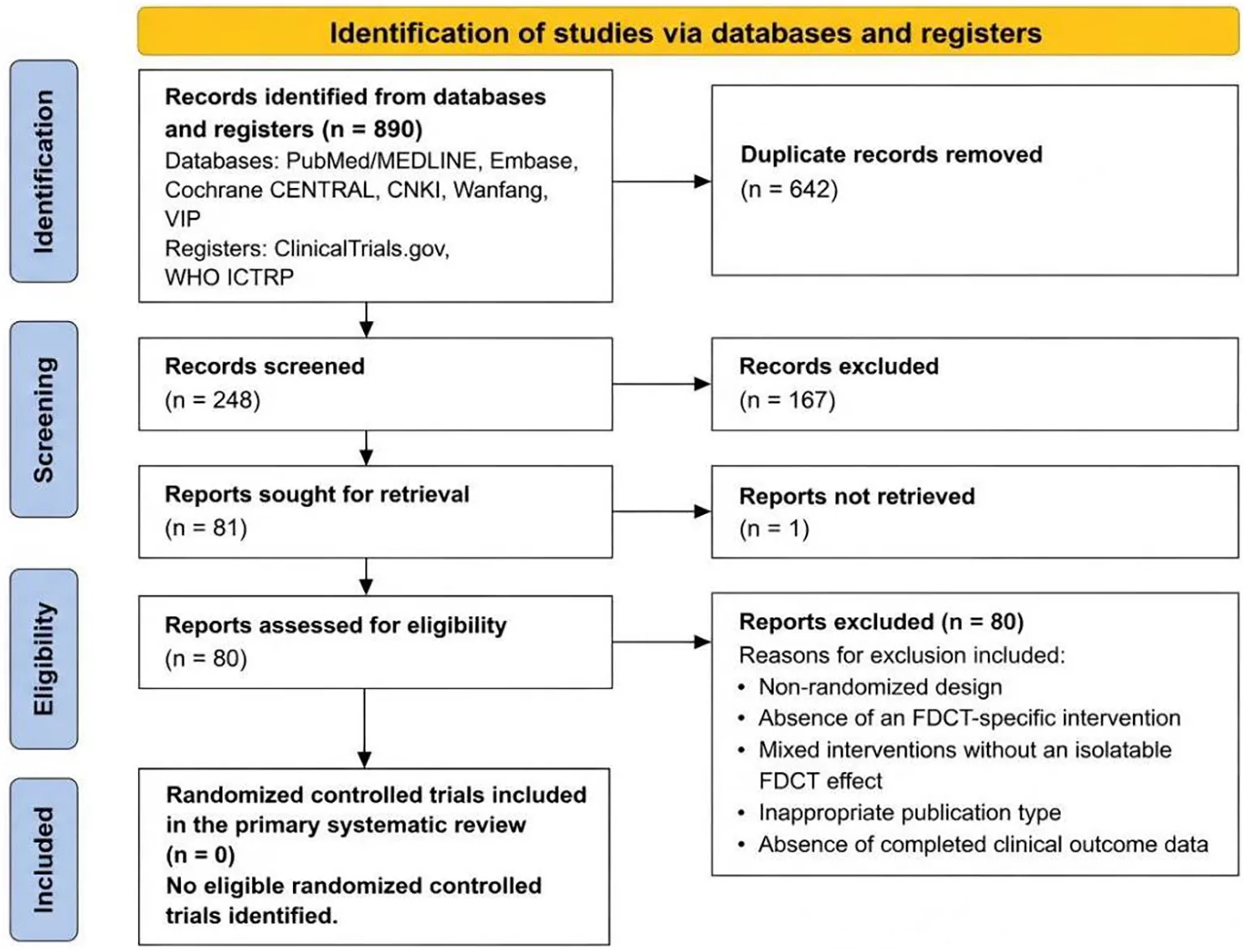
PRISMA 2020 flow diagram of study selection for the primary systematic review. No randomized controlled trial met the eligibility criteria; direct non-randomized and indirect sources were retained only for separate contextual analysis.

### 3.2 Characteristics of direct and contextual evidence

The available direct FDCT literature was heterogeneous in study design, intervention reporting, comparators, and outcome measurement. The most informative direct evidence was a retrospective comparative study of 112 women with PD classified as cold coagulation and blood stasis syndrome: 58 received FDCT combined with Shaofu Zhuyu Decoction and 54 received Shaofu Zhuyu Decoction alone (15). Treatment was administered over three consecutive menstrual cycles. Two additional reports described FDCT or dragon-cupping interventions but provided insufficient methodological and numerical detail for reliable causal interpretation (7, 13). Other contextual sources evaluated conventional dry or static cupping, moxibustion, acupuncture-related procedures, herbal treatment, or other multimodal TCM approaches. Because these interventions differed from FDCT in thermal exposure, suction technique, movement, acupoint selection, treatment intensity, comparator, and co-interventions, they were treated as indirect evidence rather than combined with the direct FDCT literature. Essential characteristics of the direct clinical studies are summarized in **Table 1**.

**Table 1:** Characteristics of direct clinical studies evaluating fire dragon cupping therapy for primary dysmenorrhea.

Study title	Reference	Design	Sample	Intervention and comparator	Key outcomes	Limitations
The efficacy of Fire Dragon cupping combined with Shaofu Zhuyu decoction in treating primary dysmenorrhea with cold coagulation and blood stasis	Yang et al. (2025) (15)	Retrospective comparative	112 (58 vs 54)	FDCT + Shaofu Zhuyu Decoction vs Shaofu Zhuyu Decoction alone	Lower post-treatment pain and TCM syndrome scores, RI, PI, and ET-1; higher NO; reported between-group comparisons P < 0.05	Non-randomized allocation; active herbal co-intervention; FDCT-specific effect cannot be isolated
Clinical observation of Fire Dragon cupping therapy in primary dysmenorrhea	Wang et al. (2021) (7)	Observational	NR	FDCT; comparator NR	Pain/symptom improvement reported	Protocol, comparator, and numerical data insufficient for reliable causal interpretation
Effect of dragon cupping therapy on primary dysmenorrhea	Zhong et al. (2021) (13)	Non-randomized	NR	Dragon/FDCT-based cupping; comparator NR	Pain/symptom improvement reported	Intervention, comparator, and outcome details insufficient for reliable causal interpretation

FDCT, Fire Dragon Cupping Therapy; TCM, Traditional Chinese Medicine; VAS, Visual Analog Scale; RI, resistance index; PI, pulsatility index; ET-1, endothelin-1; NO, nitric oxide; NR, not reported.

### 3.3 Pain and symptom outcomes

In the retrospective comparative study, the FDCT-plus-Shaofu Zhuyu Decoction group had lower post-treatment VAS pain scores and TCM syndrome scores than the decoction-only group, with all reported between-group comparisons reaching P < 0.05 (15). The less completely reported direct studies also described improvement in pain or dysmenorrhea-related symptoms (7, 13). Several contextual studies of conventional dry cupping and other TCM interventions reported reductions in menstrual pain (10, 16–22). However, differences in interventions, study designs, comparators, and outcome reporting precluded quantitative synthesis across these sources.

### 3.4 Hemodynamic and biochemical outcomes

Uterine artery RI and PI and serum ET-1 and NO were reported in the retrospective comparative study (15). Compared with the Shaofu Zhuyu Decoction-only group, the combined-treatment group had lower post-treatment RI and PI, lower ET-1, and higher NO, with all reported between-group comparisons reaching P < 0.05. These outcomes describe the between-group associations reported in that study and were not used to infer an independent FDCT-specific physiological effect (15).

### 3.5 Safety and tolerability outcomes

Safety information was incomplete and inconsistently reported across the available direct and contextual evidence. Reported adverse effects were predominantly mild and transient local reactions associated with cupping-based procedures, including erythema, warmth, discoloration, and cupping marks (8, 14). Serious adverse events were not reported in the available sources. Most reports, however, did not provide prespecified adverse-event definitions, systematic surveillance, severity grading, treatment-relatedness assessment, withdrawals due to adverse events, or extended post-treatment follow-up.

### 3.6 Risk of bias and certainty of evidence

The most informative direct retrospective study was judged to have a serious overall risk of bias, particularly because of non-random treatment allocation, possible confounding, lack of blinding, use of an active herbal co-intervention, and limited safety ascertainment. The other direct reports were incompletely described, further restricting methodological assessment. Certainty was considered very low for pain and symptom outcomes, uterine hemodynamic measures, biochemical markers, and safety.

Taken together, the available studies provide preliminary, hypothesis-generating observations rather than a basis for comparative-effectiveness estimates or subgroup analyses. An overview of the evidence synthesis process and principal findings is presented in **Figure 2**.

**Figure 2:**
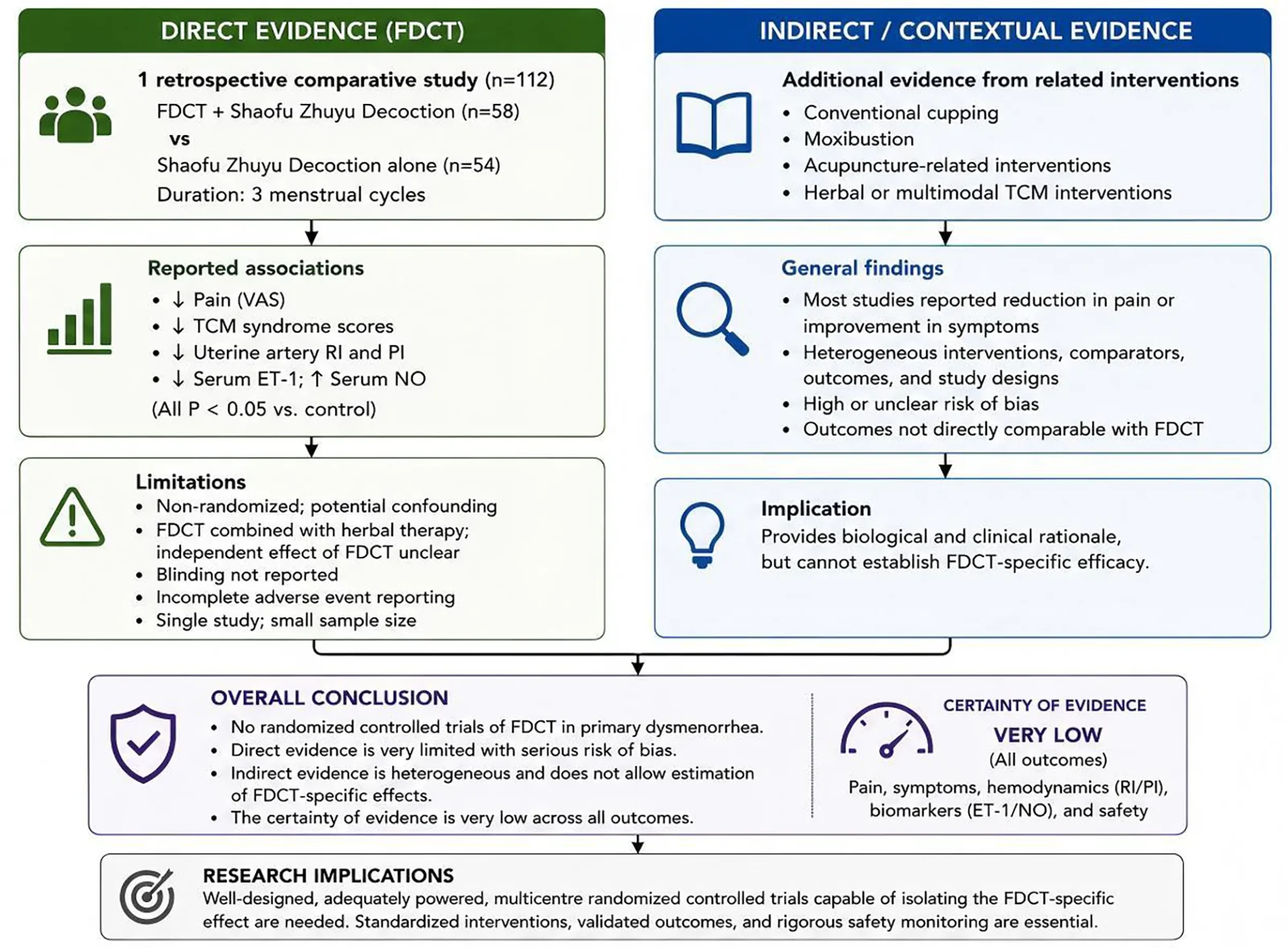
Framework of direct and indirect clinical evidence for FDCT in primary dysmenorrhea. Direct evidence was limited to non-randomized clinical evidence, whereas evidence from related interventions was considered indirect or contextual. Reported findings from combination therapy cannot establish an independent FDCT-specific treatment effect. Overall certainty of evidence was considered very low, and the proposed clinical and biological implications should be interpreted as hypothesis-generating.

## 4 Discussion

This systematic review identified a substantial evidence gap in the evaluation of FDCT for PD. The predefined search did not identify an eligible RCT, and the available literature therefore cannot provide a reliable randomized estimate of treatment effect, comparative effectiveness, durability, or safety. This distinction is important because clinical use or biological plausibility should not be interpreted as evidence of efficacy (14–18).

The principal direct comparative evidence was a retrospective study of FDCT administered with Shaofu Zhuyu Decoction (15). Its findings are clinically relevant for hypothesis generation, but routine-practice treatment selection, non-random allocation, and the active co-intervention limit causal interpretation. Evidence from conventional dry cupping, moxibustion, acupuncture-related procedures, and other multimodal TCM interventions may inform the rationale and design of future FDCT-specific trials, but these therapies differ from FDCT in thermal exposure, suction, movement, acupoint selection, intensity, and co-interventions (8, 10, 16–18, 23–25).

The reported changes in uterine artery RI and PI and in ET-1 and NO warrant further investigation as exploratory physiological endpoints (11, 15). They should not, however, be regarded as established mechanisms of FDCT. Proposed analgesic, anti-inflammatory, circulatory, and neurovascular pathways remain hypotheses derived largely from related therapies and physiological reasoning and require confirmation in rigorously designed FDCT-specific studies.

Safety requires similarly rigorous evaluation. The available reports mainly described transient local skin reactions and did not report serious adverse events (8, 14). But surveillance methods and follow-up were insufficient to characterize uncommon, delayed, or cumulative harms. Future trials should therefore integrate prospective adverse-event definitions, severity grading, attribution, and follow-up into the protocol rather than relying on passive reporting.

Accordingly, the present evidence supports further investigation of FDCT rather than routine substitution for established PD treatments. Future research should prioritize intervention standardization, clinically meaningful patient-reported outcomes, appropriate comparators, and transparent safety monitoring (25).

## 5 Clinical implications

FDCT cannot currently be recommended as an evidence-based replacement for NSAIDs, hormonal therapy, or other established approaches. For patients who choose FDCT as a complementary intervention, clinicians should explain the limitations of the evidence, individualize care, ensure delivery by appropriately trained practitioners, and maintain standard diagnostic assessment and evidence-based management. The favorable pain, hemodynamic, and biomarker findings reported in the retrospective comparative study (15) should be interpreted as hypothesis-generating and should not be used to claim superiority over conventional therapy or modification of the underlying disease process.

## 6 Strengths and limitations

A strength of this review was the broad search of international, Chinese, and trial-registry databases and the explicit attempt to distinguish FDCT from conventional cupping and other TCM interventions. The revised synthesis separately categorized eligible randomized evidence, direct non-randomized evidence, indirect clinical evidence, reviews, and ongoing trials, thereby reducing the risk that methodologically different sources would be interpreted as equivalent.

This review also has important limitations. Direct clinical evidence was limited to non-randomized and incompletely reported studies, and the most informative study evaluated FDCT as part of a combined intervention, preventing estimation of an independent FDCT effect. Intervention protocols, diagnostic criteria, outcome measures, treatment durations, and follow-up periods varied across contextual sources. Quantitative reviewer agreement was not prospectively recorded, and publication bias could not be assessed, and some Chinese-language publications provided limited methodological detail, and incomplete reporting restricted risk-of-bias assessment. These limitations restrict causal interpretation and reinforce the need for prospective FDCT-specific trials.

## 7 Future research directions

Future studies should use prospective, adequately powered, multicenter randomized controlled designs. Sample-size calculations should be based on a prespecified clinically important difference in validated pain scores. Trials should distinguish FDCT used alone from FDCT used as an adjunct and should include appropriate comparators such as sham thermal cupping, sham suction, usual care, non-steroidal anti-inflammatory drugs, hormonal therapy, or the same co-intervention without FDCT.

A standardized FDCT protocol should define cup design, source and intensity of heat, target skin-temperature range, suction pressure, anatomical sites and acupoints, moving-cup repetitions, session duration, treatment frequency, timing in relation to menstruation, number of menstrual cycles, practitioner qualifications, and criteria for treatment modification or discontinuation. Treatment fidelity should be documented using procedural checklists, temperature monitoring, session attendance, and independent protocol auditing.

Core outcomes should include validated pain-intensity measures, rescue-analgesic use, functional impairment, school or work absenteeism, health-related quality of life, patient global improvement, recurrence, and treatment acceptability. Hemodynamic and biochemical outcomes may be included as exploratory endpoints but should not replace clinically meaningful patient-reported outcomes. Follow-up should extend beyond the immediate treatment period, preferably for at least three to six menstrual cycles after treatment, to assess durability, recurrence, and delayed adverse events.

Safety reporting should use prespecified definitions and severity grading for burns, blistering, persistent skin changes, infection, vasovagal symptoms, discomfort, treatment discontinuation, and serious adverse events. Future studies should also report withdrawals, adherence, protocol deviations, practitioner experience, funding sources, and conflicts of interest. Prospective registration, publication of detailed protocols, and reporting according to CONSORT guidelines would improve transparency and reproducibility (22–24).

## 8 Conclusion

Current evidence is insufficient to establish the efficacy, comparative effectiveness, durability, or safety of FDCT for primary dysmenorrhea. The available direct evidence is non-randomized and includes an active herbal co-intervention, while evidence from conventional cupping and other TCM interventions is not equivalent to FDCT-specific evidence. FDCT should therefore be regarded as an insufficiently evaluated complementary intervention. Standardized, adequately powered, multicenter RCTs with validated clinical outcomes, appropriate comparators, longer follow-up, and systematic safety surveillance are required.

## Data Availability

The original contributions presented in the study are included in the article/supplementary material. Further inquiries can be directed to the corresponding author.
